# Emotion dysregulation in adolescents: the associations with clinical symptoms, risky-behaviors, and environmental factors

**DOI:** 10.1192/j.eurpsy.2024.938

**Published:** 2024-08-27

**Authors:** L. Pedrini, S. Meloni

**Affiliations:** UO Psichiatria, IRCCS Centro San Giovanni di Dio Fatebenefratelli, Brescia, Italy

## Abstract

**Introduction:**

Emotion dysregulation (ED) is transdiagnostic domain that plays a pivotal role in the emergence and persistence of numerous mental disorders. Examining the extent of ED within non-clinical populations may shed light on whether ED is indeed linked to symptoms as observed in clinical settings. This investigation constitutes a crucial milestone toward the development of preventive strategies.

**Objectives:**

To investigate the correlations between ED, psychopathological symptoms, risky behaviors, and environmental factors in adolescent students.

**Methods:**

A total of N=610 students (16 years; 72% females) completed self-report standardized questionnaires measuring depression, anxiety, impulsivity, childhood trauma, relations with classmates, and family functioning. Lifetime risky-behaviours were recorded using an ad-hoc checklist, and ED through Difficulties in Emotion Regulation Scale (DERS). The sample was then divided into subgroups based on percentiles of DERS Total scores: N=210 low ED, N=187 moderate, N=214 high.

**Results:**

Participants exhibiting high ED displayed higher level of depression, anxiety and impulsivity (Table 1). There was an observable trend linking higher levels of ED with a greater proportion of youths reporting risky behaviors (Table 2). The high ED group reported an increased frequency of childhood traumatic experiences, less favorable relationships with family members and classmates (Table 3).

**Table 1.** Clinical symptoms by level of ED in students (N=610)

	low	moderate	high	Sig.
Patient Health Questionnaire (depression)	5.33(±3.51)	8.94(±4.05)	14.57(±5.53)	<.001
Screen For Child Anxiety Related Emotional Disorders (anxiety)	59.27(±9.61)	68.61(±10.88)	79.39(±11.61)	<.001
Barratt Impulsiveness Scale-Brief (impulsivity)	15.14(±3.52)	16.70(±3.81)	18.01(±4.17)	<.001

**Table 2.** Risky behaviors by level of ED in students (N=610)

	low	moderate	high	Sig.
Binge drinking	N=73 (29.6%)	N=78 (31.6%)	N=96 (38.9%)	.097
Self-harm ideation	N=35 (13.4%)	N=73 (28%)	N=153 (58.6%)	<.001
Self-harm	N=30 (15%)	N=49 (24.5%)	N=121 (60.5%)	<.001
Binge eating	N=60 (22.9%)	N=78 (29.8%)	N=124 (47.3%)	<.001

**Table 3.** Environmental factors by level of ED in students (N=610)

	low	moderate	high	Sig.
Childhood Trauma Questionnaire (trauma)	30.99(±6.89)	35.39(±9.1)	39.54(±10.94)	<.001
Child And Adolescent Social Support Scale (classmate)	51.19(±11.7)	46.55(±10.96)	44.91(±12.4)	<.001
Family Assessment Device (family functioning)	117.58(±14)	108.8(±17.48)	103.38(±20.11)	<.001

**Conclusions:**

Findings provide robust support for the association between ED and compromised personal functioning, even within a non-clinical sample. The trend observed in the relationship between ED, clinical symptoms and risky behaviors is consistent across all variables. Overall, these results contribute to the growing body of evidence advocating for preventive interventions aimed at addressing ED in adolescents.

**Disclosure of Interest:**

None Declared

